# B Cell, Th17, and Neutrophil Related Cerebrospinal Fluid Cytokine/Chemokines Are Elevated in MOG Antibody Associated Demyelination

**DOI:** 10.1371/journal.pone.0149411

**Published:** 2016-02-26

**Authors:** Kavitha Kothur, Louise Wienholt, Esther M Tantsis, John Earl, Sushil Bandodkar, Kristina Prelog, Fiona Tea, Sudarshini Ramanathan, Fabienne Brilot, Russell C. Dale

**Affiliations:** 1 Neuroimmunology group, Institute for Neuroscience and Muscle Research, The Children’s Hospital at Westmead, University of Sydney, Sydney, NSW, Australia; 2 Department of Clinical Immunology, Royal Prince Alfred Hospital, Sydney, NSW, Australia; 3 Department of Biochemistry, The Children’s Hospital at Westmead, Sydney, NSW, Australia; 4 Department of Medical Imaging, The Children’s Hospital at Westmead, Sydney, NSW, Australia; LMU Munich, GERMANY

## Abstract

**Background:**

Myelin oligodendrocyte glycoprotein antibody (MOG Ab) associated demyelination represents a subgroup of autoimmune demyelination that is separate from multiple sclerosis and aquaporin 4 IgG-positive NMO, and can have a relapsing course. Unlike NMO and MS, there is a paucity of literature on immunopathology and CSF cytokine/chemokines in MOG Ab associated demyelination.

**Aim:**

To study the differences in immunopathogenesis based on cytokine/chemokine profile in MOG Ab-positive (POS) and -negative (NEG) groups.

**Methods:**

We measured 34 cytokines/chemokines using multiplex immunoassay in CSF collected from paediatric patients with serum MOG Ab POS [acute disseminated encephalomyelitis (ADEM = 8), transverse myelitis (TM = 2) n = 10] and serum MOG Ab NEG (ADEM = 5, TM = 4, n = 9) demyelination. We generated normative data using CSF from 20 non-inflammatory neurological controls.

**Results:**

The CSF cytokine and chemokine levels were higher in both MOG Ab POS and MOG Ab NEG demyelination groups compared to controls. The CSF in MOG Ab POS patients showed predominant elevation of B cell related cytokines/chemokines (CXCL13, APRIL, BAFF and CCL19) as well as some of Th17 related cytokines (IL-6 AND G-CSF) compared to MOG Ab NEG group (all p<0.01). In addition, patients with elevated CSF MOG antibodies had higher CSF CXCL13, CXCL12, CCL19, IL-17A and G-CSF than patients without CSF MOG antibodies.

**Conclusion:**

Our findings suggest that MOG Ab POS patients have a more pronounced CNS inflammatory response with elevation of predominant humoral associated cytokines/chemokines, as well as some Th 17 and neutrophil related cytokines/chemokines suggesting a differential inflammatory pathogenesis associated with MOG antibody seropositivity. This cytokine/chemokine profiling provides new insight into disease pathogenesis, and improves our ability to monitor inflammation and response to treatment. In addition, some of these molecules may represent potential immunomodulatory targets.

## Introduction

Myelin oligodendrocyte glycoprotein (MOG) is a myelin antigen located at the outer surface of the central nervous system (CNS) myelin sheath, and is a target for autoimmune responses that results in CNS inflammation and demyelination [1]. Using cell-based assays, serum MOG antibody has been shown to be primarily associated with pediatric acute disseminated encephalomyelitis (ADEM), optic neuritis (ON), transverse myelitis (TM), and aquaporin 4 antibody-negative neuromyelitis optica (NMO) [2–4]. In adults, MOG antibody is predominantly seen in association with NMO/NMOSD, including bilateral and/or recurrent optic neuritis, and transverse myelitis, but rarely seen in patients with MS [5–8]. While some studies evaluating paediatric patients showed a persistence of MOG antibody seropositivity with relapsing disease, the role of MOG antibody as a biomarker for disease recurrence or severity remains to be clarified [9, 10]. Relapsing MOG Ab demyelination syndromes are considered distinct from multiple sclerosis (MS) based on clinical and neuroimaging features, and MOG antibody seropositivity is associated with a non-MS course at 1-year follow-up [11, 12].

There is emerging evidence that MOG antibodies are pathogenic in human demyelinating diseases. *In vitro* experiments have shown that patient serum containing MOG antibodies can induce complement, natural killer cell and antibody dependent cell mediated toxicity, and disrupt oligodendrocyte cytoskeleton [2, 13–16]. NMO associated with aquaporin-4 antibodies is an astrocytopathy and there are a large number of studies demonstrating a complex immunopathology involving B cells, eosinophils, neutrophils, and Th17 cell mechanisms [17–19]. By contrast, other than autoantibody-associated mechanisms, there is limited information available on the immune-pathogenesis of MOG antibody-mediated inflammation. Likewise, although the presence of different CSF cytokines and chemokines has previously been investigated in NMO [20], CSF cytokine studies in MOG-associated demyelination are lacking.

Cytokines/chemokines are biologically active polypeptide intercellular messengers that have pleiotropic effects on a variety of cell types leading to immune system activation, including proliferation, differentiation, and recruitment of immune cells to site of inflammation contributing to immunopathogenesis [21]. There is a considerable overlap and redundancy between cytokines/chemokines with respect to individual functions, despite some unique properties. CD41 naive T cells can differentiate into distinct subsets (Th1, Th2, Th17, Th22, and T-follicular effector cells) depending on the nature of antigen and surrounding cytokine milieu which inturn causes inflammation through cytokine and chemokine reponses [21–23].

In view of emerging evidence of pathogenicity of MOG antibodies, we hypothesize that MOG Ab POS demyelination has a dominant humoral immune pathogenesis compared to MOG Ab NEG group. In this study, we investigated and analysed a broad array of CSF cytokines/chemokines in MOG Ab POS and NEG groups, and their association with clinical/laboratory findings.

## Patients and Methods

### Patients and Controls

As part of the ethically approved protocol, we wrote to all parents and gained written consent to use the stored CSF for this study. This study was approved by HREC of Sydney Children’s Hospital network (LNRSSA/14/SCHN/283). We selected patients with a first episode of acute CNS demyelinating disease who had stored frozen acute CSF available, using Neurology and Neuroimmunology clinical databases at the Children's Hospital at Westmead, Sydney, Australia. All the CSF samples in controls and patients were collected over a 7 yr period from 2007–2014 except for 2 controls in whom CSF was collected between 2001 and2002. Patients were classified according to MOG antibody serostatus on stored acute sera using a fluorescence-activated cell sorting (FACS) live cell-based assay. We detected antibody binding of patient serum and CSF IgG to human full-length surface MOG transduced in HEK293 cells as previously described [2, 7]. Samples were considered positive if they were above threshold on at least 2 of 3 repeated experiments.

A total of 10 patients with MOG Ab POS [acute disseminated encephalomyelitis (ADEM) = 8, transverse myelitis (TM) = 1, and TM + optic neuritis (ON) = 1] and 9 patients with MOG Ab NEG (ADEM = 5 and TM = 4) demyelination were included. We ensured that MOG Ab POS and NEG groups were clinically similar with regard to proportions of ADEM and TM. Patients with ADEM and TM fulfilled International Pediatric MS Study Group (IPMSSG) diagnostic criteria 2013 [24]. Twenty non-inflammatory neurological controls were used to generate a reference range, and included cerebral palsy (n = 8) [kernicterus (2), extreme prematurity (1), placental insufficiency (1), unknown cause (4)], neurotransmitter disorders (n = 4) [Dopa responsive dystonia (3), and 6PTPS deficiency (1)], monogenic movement disorders (n = 6), stereotypy (n = 1), and congenital myesthenic syndrome (n = 1). All CSF samples were collected during routine diagnostic work-up, prior to commencing treatment, and samples were frozen at -40 degrees until analysis. The clinical features and CSF findings are presented in brief in Table 1.

**Table 1:** Clinical and laboratory investigations in MOG Ab POS and NEG demyelination syndromes.

	MOG Ab POS (n = 10)	MOG Ab NEG (n = 9)	Control (n = 20)
Clinical syndrome (n)	ADEM (8), TM (1), TM+ON (1)	ADEM (5), TM (4)	NIND
Age, median (range), y	4.7 (2–14) ^a^	9.3 (5.5–13)	4.9 (0.3–14)*
Timing of CSF from onset of neurological symptoms, median (range), d	6 (2–15)	4 (2–7)	N/A
Elevated ESR (>20mm/hr)(proportion)	6/9	2/5	N/A
CSF Pleocytosis, median (range), 10^6^ /L	33 (0–175)	17.5 (0–66)	0 (0–3)
CSF neutrophils, median (range), 10^6^ /L	5 (0–25)	1 (0–6)	0 (0–1)
CSF protein, median (range), mg/dl	0.42 (0.15–0.71)	0.29 (0.11–0.70)	0.25 (0.1–0.78)
CSF IgG, median (range), g/L (n)	0.04 (0.026–0.08) ^b^ (n = 7)	0.025 (0.009–0.04) (n = 6)	N/M
CSF IgG/albumin, median (range)# (n)	18 (13–27)(n = 7)	14 (11–23) (n = 5)	N/M
FU outcome (MRS score)			N/A
No disability (0–1)	4	6	
Mild disability (2)	5	2	
Moderate disability (3)	1	1	
Duration of FU, mean (SD), mo	20.8 (19.1)	22.8 (18.8)	

**Abbreviations**: MOG, myelin oligodendrocyte glycoprotein; Ab, antibody; POS, positive; NEG: negative; ADEM, acute disseminated encephalomyelitis; TM, transverse myelitis; ESR, erythrocyte sedimentation rate; y, years; d, day; mo, months: CSF, cerebrospinal fluid, MRS, modified Rankin scale; FU, follow up; NIND, non-inflammatory neurological disorders; N/A, not applicable; N/M, not measured

^#^ Normal CSF IgG/albumin = <10;

* Age of CSF sampling

^a^ p = 0.04

^b^ p = 0.005, p value was calculated using Mann-Whitney test

#### MRI

The radiological abnormalities on acute and follow up MRI brain including extent and configuration of lesions were recorded and rated by neuroradiologist (KP) blinded to diagnosis according to the previously published protocol by Baumann et al [25]. The sequences reviewed included T2 axial, T2 fluid attenuated inversion recovery-coronal, T1-sagittal, T1-axial with gadolinium (GAD), spinal cord T2 axial, sagittal and T1-sagittal with GAD.

#### Multiplex cytokine/chemokine immunoassay

Thirty-two cytokines were measured by multiplexed fluorescent bead-based immunoassay detection (MILLIPLEX^®^ MAP system, Millipore Corporation, Missouri U.S.A.) according to the manufacturer’s instructions, using a combination of 23-plex (MPHCYTOMAG60K23), 6 plex (MPHCYP2MAG62K06), and 3-plex (MPHCYP3MAG63K03) Millipore Human Cytokine panel kits. The 23-plex kit contained antibody-conjugated beads for following cytokines and chemokines: IL-1ra, GM-CSF, IL-1b, TNF-α, IL-2, IL-4, IL-6, IL-8, IL-10, IL-13, IL-17A, IFN-γ, CCL2/MCP-1, CCL5/RANTES, CXCL1/GRO, CXCL10/IP-10, CCL3/MIP-1a, CCL4/MIP-1b, IL-12 (p40) and IL-12 (p70), IFN-α, G-CSF and CCL11/Eotaxin. The 6-plex kit was used to detect IL-21, IL-23, CXCL13/BCA-1, CCL17/TARC, CCL21/6Ckine and CXCL12/SDF-1. The 3 Plex kit contained antibody-conjugated beads for CXCL9/MIG, CXCL11/I-TAC, and CCL19/MIP-3b. For each assay, the curve was derived from various concentrations of the cytokine standards assayed in the same manner as patient samples. All samples were measured undiluted. The methodological details including assay method, lower detection limits and coefficient of variance are available at the manufacturer’s website, “www.merckmillipore.com”

### Enzyme-linked immunosorbent assay (ELISA)

APRIL (sensitivity <2pg/ml) and BAFF (sensitivity 0.4ng/ml) were performed using ELISA kits as per the manufacturer’s instructions (R&D Systems, Minneapolis, MN). All samples were analyzed undiluted except 6 samples, where volume was insufficient for BAFF analysis. In these cases, results were multiplied by the appropriate dilution factor.

### Statistical analysis

Statistical analysis was performed using R statistical programme and graphs were composed using Graph Pad Prism software version 6. We compared clinical and demographic features between MOG Ab POS and NEG groups using Mann-Whitney test. The CSF cytokine/chemokine data were analyzed to check for a normal distribution. As data did not show Gaussian distribution, statistical analyses of cytokine/chemokine levels were performed using Kruskal-Wallis test for multiple groups and Mann-Whitney test for two groups and 2 tailed p value was calculated. No adjustment was made for multiple group statistical comparisons, as this study was largely exploratory with an intention to study the immunopathological mechanisms based on CSF cytokines/chemokines. The p-values from these analyses should be viewed as a measure of the strength of evidence for each association. We examined the correlations between cytokine/chemokine levels in the CSF and with disability score during admission and follow up by the Spearman rank correlation test. In the correlation analysis, p values <0.05 were considered to indicate statistical significance.

## Results

### Comparison of clinical and radiological features (MRI) between MOG Ab POS and MOG Ab NEG demyelination groups

The patients in MOG Ab POS and control group were younger compared to MOG Ab NEG group (median 4.7 vs 4.9 vs 9.3 years). The time to CSF sampling from onset of neurological symptoms was shorter in the MOG Ab NEG group compared to MOG Ab POS group (median 4 vs 6 days). The ESR was more commonly elevated in MOG Ab POS group. The CSF cell count (median 33 vs 17.5 10^6^ /L), CSF neutrophils (median 5 vs 1 10^6^ /L), CSF IgG (median 0.04 vs 0.025 g/L), and protein (median 0.42 vs 0.29 mg/dl) levels were higher in the MOG Ab POS CSF samples than in the MOG Ab NEG (Table 1). Recurrent relapses occurred in 2/10 of the MOG Ab POS patients, 8 month and 3 years after the initial events, whereas only 1/9 patient in the MOG Ab NEG group relapsed once within 2 months of the illness. There were no significant differences in disability outcome. The details on treatment and outcome are given in Table A in S1 File.

**Fig 1:**
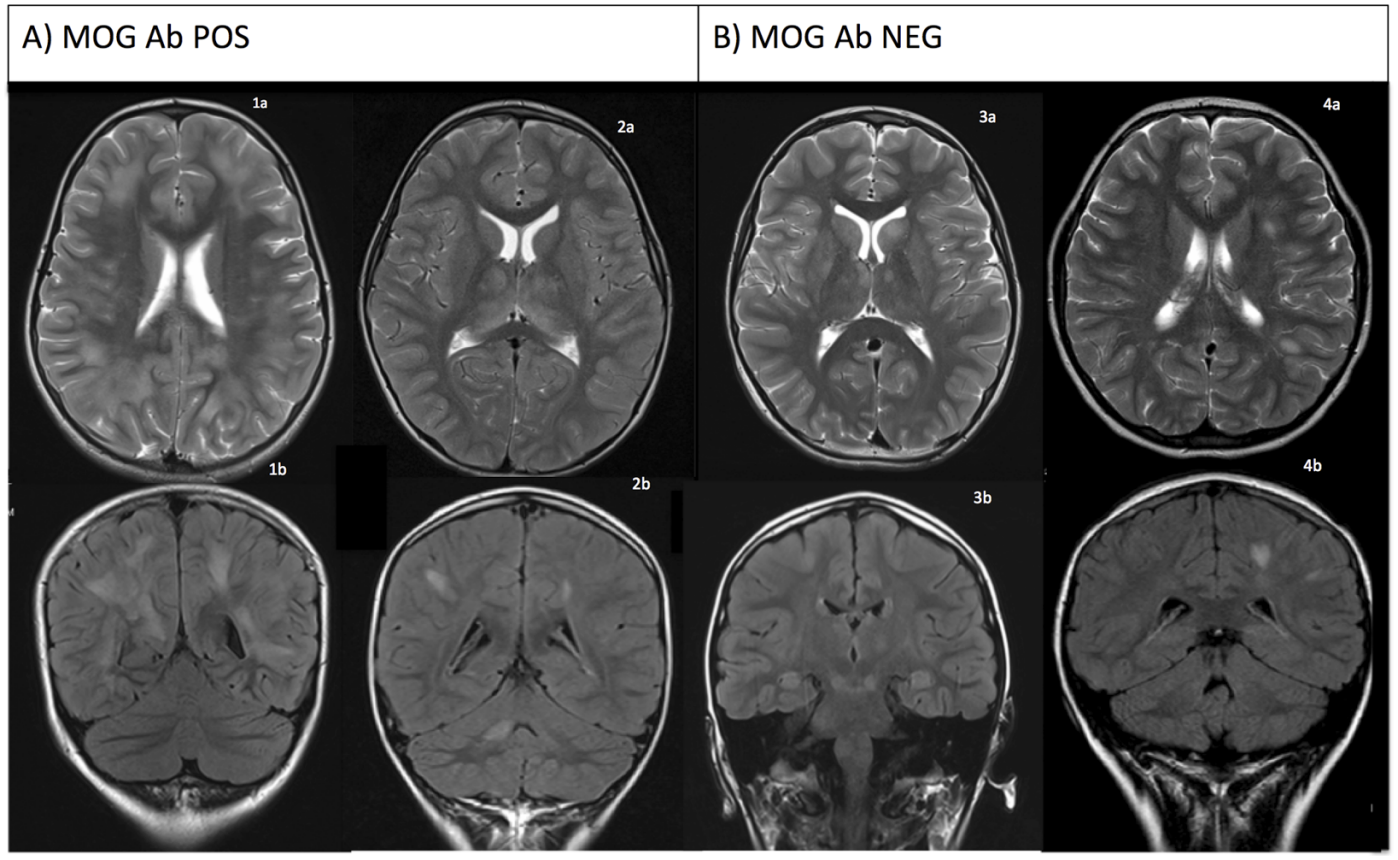
Comparison of MRI brain radiological features in serum MOG Ab POS vs MOG Ab NEG demyelination group. **A) MOG Ab POS group**: (a) T2 axial and (b) T2 FLAIR coronal views of case 1 show large subcortical demyelinating lesions. Case 2 shows confluent bilateral thalamic lesions and subcortical diffuse demyelinating lesions with hazy margins. **B) MOG Ab NEG group**: (a) T2 axial and (b) T2 FLAIR coronal views of case 3 show small multiple hyperintensities in putamina, head of caudate nuclei, thalami, midbrain, posterior pons, and medulla. Case 4 shows small well-defined demyelinating lesions in left subcortical white matter.

The MRI brain in the MOG Ab POS group showed more extensive white matter involvement (mean supratentorial white matter score 3.2 vs 1.8), larger white matter lesion size, contrast enhancement of lesions, T1 hypointense lesions, optic nerve involvement, and more residual signal changes at follow up (FU) MRI compared to MOG Ab NEG group (Fig 1 and **Table B in** S1 File).

### Comparison of CSF cytokine/chemokine levels between MOG Ab POS, MOG Ab NEG demyelination groups and controls

Table 2 presents the median and ranges for all CSF cytokine/chemokines and statistical comparisons, presented according to Th1, Th2, Th17, Treg, B cell, and other cytokines/chemokines. 27 of the 34 measured CSF cytokines and chemokines were elevated in MOG Ab POS group compared to controls. Likewise, 24 of the 34 measured CSF cytokines/chemokines were elevated in MOG Ab NEG group compared to controls.

**Table 2:** CSF cytokine/chemokine concentrations (median and range) in MOG Ab POS, MOG Ab NEG demyelination groups and controls according to T and B cell subsets. This table shows predominant elevation of B cell and some of Th17 cytokines/chemokines in MOG Ab POS demyelination compared to MOG Ab NEG group.

Cytokines/chemokines (pg/ml)	MOG Ab POS	MOG Ab NEG	Control	MOG Ab POS vs Control	MOG Ab NEG vs Control	MOG Ab POS vs MOG Ab NEG
	Median (range)			p values		
**Th1**						
IFN-γ	3.3 (0.6–16.70)	0 (0–63.9)	0 (0)	<0.0001***	0.002**	0.1
TNF-α	6 (1.7–7.1)	1 (1.2–2.9)	0 (0–0.1)	<0.0001***	<0.0001***	0.02*
CXCL9/MIG	150.3 (53.7–551.6)	144 (25.7–680.3)	26.9 (13.4–143.7)	<0.0001***	0.005**	0.37
IP-10/CXCL10	2940.7 (646.5–8292)	1425.2 (181.9–12070)	666.2 (327.4–985.6)	0.0003***	0.28	0.25
CXCL11	3.6 (0–11)	3.6 (0.4–23.5)	5.1 (0.4–8.2)	0.44	0.89	0.51
**Th2**						
IL-2	0.2 (0–4)	0 (0–17.1)	0.9 (0–2.9)	0.43	0.07	0.41
IL-4	9.3 (5.4–25.3)	7.4 (0–55.1)	0 (0–23.7)	<0.0001***	0.0003***	0.2
IL-13	2.9 (2.3–9.4)	2.3 (0–12.1)	0 (0)	<0.0001***	<0.0001***	0.19
CCL17	4.9 (1–22.7)	2.2 (0–20.9)	0.4 (0–2.2)	<0.0001***	0.002**	0.18
Eotaxin	0 (0–122.3)	0 (0–115.9)	0 (0)	0.04*	0.008**	0.53
**Th17**						
IL-6	171 (5–745.4)	9.2 (1.5–19.5)	0.3 (0–5.6)	<0.0001***	0.0004***	0.007**
G-CSF	562.4 (25.4–7379)	34.2 (1.7–104)	15.4 (5.9–36.2)	<0.0001***	0.89	0.005**
GM-CSF	2.4 (0.5–6)	0.5 (0.5–38)	0.2 (0–4.1)	0.006**	0.25	0.05
IL-8	254.3 (26.9–516.7)	129 (6.5–366.1)	32.4 (19.6–77.3)	0.0002***	0.003**	0.19
IL-17A	1.6 (0–10.2)	0.2 (0–98)	0 (0)	<0.0001***	0.0003***	0.08
IL-23	10.1 (0–127.3)	10.1 (0–2276)	0 (0)	<0.0001***	0.0003***	1
**B cell**						
MIP3b/CCL19	1186.7 (305.4–2599)	130.4 (82.40–1224)	108.8 (37.7–320.6)	<0.0001***	0.047*	0.005**
APRIL	5.9 (3–9.2)	2.6 (1.4–4.7)	3.06 (1.4–5.2)	<0.0001***	0.31	0.0009***
BAFF	368.3 (0–3029)	90.62 (0–366.4)	227.5 (142–647)	0.06	0.01*	0.01*
BCA.1/CXCL13	329.8 (14.4–1000)	6.8 (0–89.5)	1.8 ((0–8.4)	<0.0001***	0.01*	0.003**
CXCL12	1746.3 (806.1–3701)	1136 (542.1–2026)	1543.8 (981.5–3117)	0.4	0.01*	0.04*
**T reg**						
IL-10	5.8 (0.8–10.5)	1.6 (0–12.5)	0 (0–1.3)	<0.0001***	<0.0001***	0.04*
**Others**						
X6Ckine/CCL21	0 (0–197.2)	0 (0–1457)	0 (0–64)	0.59	0.16	0.37
IL-21	7.4 (0–12.4)	8.7 (0–36.8)	0 (0–6.5)	<0.0001***	0.0004***	0.65
GRO/CXCL1	138.1 (0–793.1)	40.7 (0–405.6)	0 (0–97.40)	<0.0001***	0.004**	0.14
IFN-α	27.2 (16.8–77.3)	19.5 (7.8–128.7)	0 (0–25.4)	<0.0001***	<0.0001***	0.18
IL-12 (p40)	11.9 (4.9–38.4)	4.9 (0–129.0)	0 (0–26.1)	0.0003***	0.003**	0.19
IL-12 (p70)	0 (0–12.3)	0 (0–28.4)	0.7 (0–5.1)	0.21	0.01*	0.42
IL-1ra	61.5 (9.2–481.3)	10.2 (1.3–101.6)	3.3 (0–20.1)	<0.0001***	0.046*	0.03*
IL-1β	1.1 (0–7.6)	0 (0–20.7)	0 (0–1.9)	0.0003***	0.29	0.05
MCP-1/CCL2	939.3 (403.8–1559)	528.5 (37.5–950)	995.2 (633–3238)	0.58	0.0006***	0.06
MIP-1α /CCL3	28.4 (0–90.9)	9.7 (0–44.8)	0 (0–22.7)	0.005**	0.18	0.12
MIP-1β/CCL4	12.9 (7.7–37.1)	6.3 (2.9–53.4)	3.5 (0–17.3)	0.002**	0.06	0.11
RANTES	18.7 (8–205.6)	18 (0.5–97.3)	0 (0–1800)	0.0002***	0.003**	0.51

**Abbreviations**: Th, T helper cell; Treg, Regulatory T cells; IL-, interleukin; IFN-, interferon; TNF, tumor necrosis factor; MIG, monokine induced by IFN-γ; IP, IFN-γ inducible protein; G-CSF, granulocyte colony stimulating factor; MIP, macrophage inflammatory protein; GM-CSF, granulocyte monocyte colony stimulating factor; APRIL, A proliferation inducing ligand; BAFF, B cell activation factor; BCA-1, B cell attracting chemokine, GRO, melanoma growth-stimulating activity alpha; MCP, monocyte chemotactic protein; RANTES, regulated on activation normal T cell expressed and secreted.

p calculated using Kruskal-Wallis test.

*P<0.05

**P<0.01

*** P<0.001

### Comparison of CSF cytokine/chemokine levels between MOG Ab POS and MOG Ab NEG groups (Table 2)

#### Th1 and Th2 related cytokines/chemokines

There was no difference between serum MOG Ab POS and MOG Ab NEG groups for CSF Th1 and Th2 molecules, except for TNF-α.

#### Th17 related cytokines and chemokines

Two of the five CSF Th17 molecules (IL-6 and G-CSF) were elevated in the serum MOG Ab POS group compared to MOG Ab NEG group.

#### B cell related cytokines and chemokines

All five of the five measured CSF B cell associated cytokines/chemokine levels were elevated in serum MOG Ab POS group compared to MOG Ab NEG group (CXCL13, CXCL12, APRIL, BAFF and CCL19).

#### Treg related cytokines and chemokines

CSF IL-10 was elevated in MOG Ab POS group compared to MOG Ab NEG group.

#### Other cytokines

Of the 13 other CSF cytokines/chemokines measured, only IL-1ra levels were higher in serum MOG Ab POS group compared to MOG Ab NEG group.

**Fig 2:**
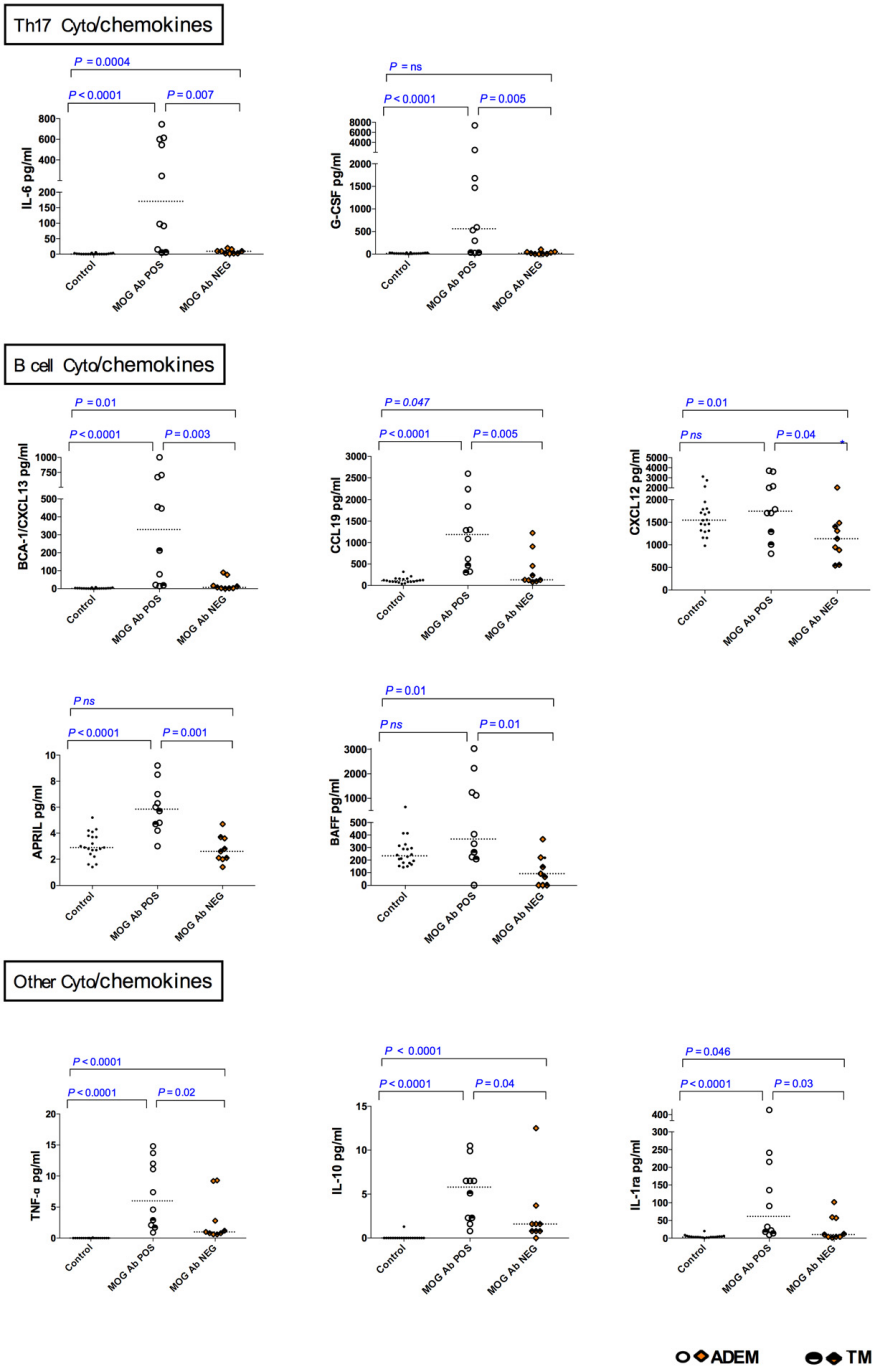
CSF cytokine/ chemokine concentrations in serum MOG Ab POS, serum MOG Ab NEG demyelination groups and controls according to T and B cell subsets. Th17 (IL-6 & G-CSF), B cell related (CXCL13, CCL19, APRIL & BAFF) and other cytokines and chemokines (TNF-α, IL1ra and IL-10) were elevated in serum MOG Ab POS patients compared to serum MOG Ab NEG group (p<0.05). Dotted lines represent medians. The statistical analysis was performed using Kruskal Wallis test.

**Fig 3:**
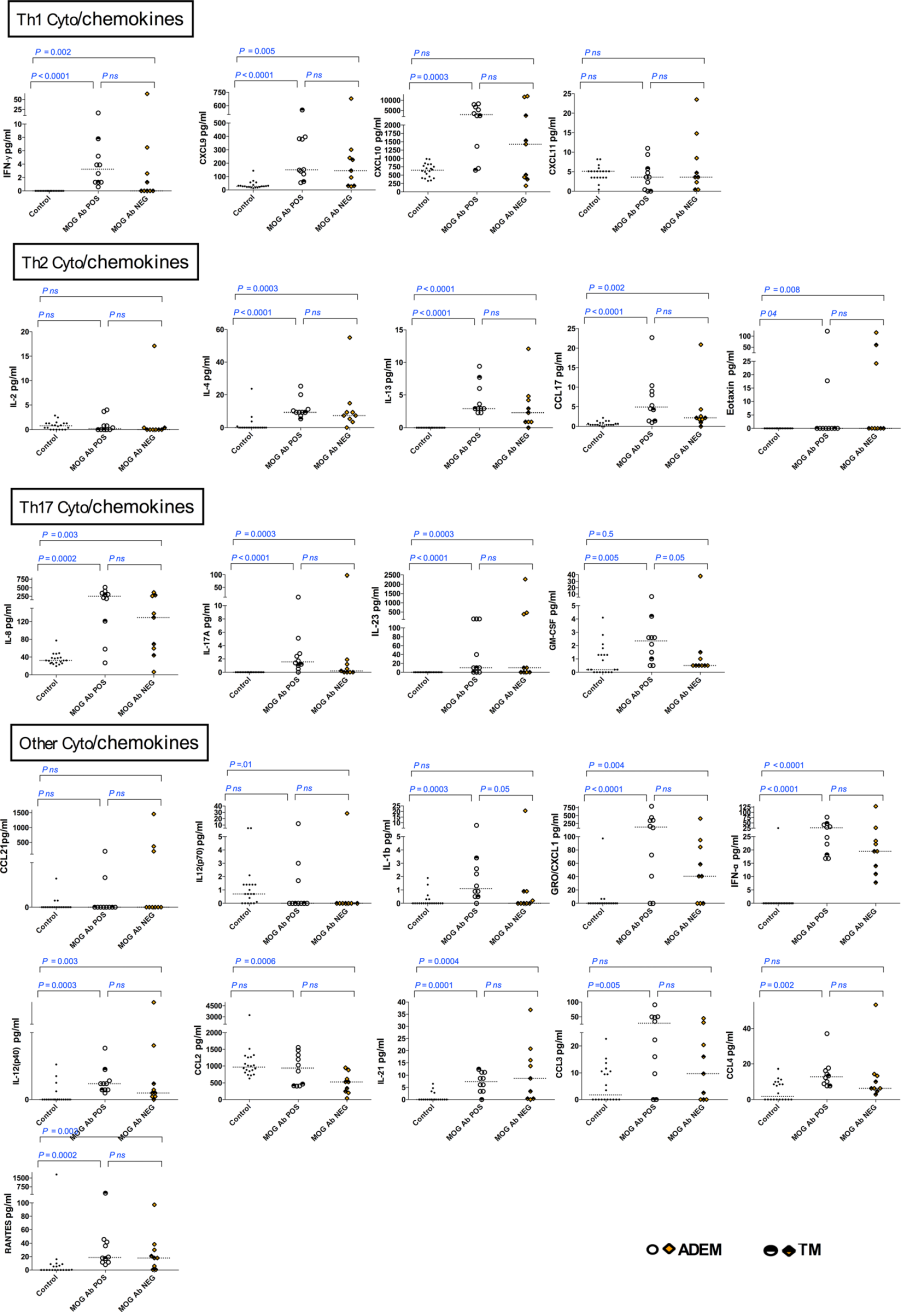
CSF cytokine/chemokine concentrations in serum MOG Ab POS, serum MOG Ab NEG demyelination groups and controls, according to T and B cell subsets. Cytokines and chemokines within Th1, Th2 and other CSF cytokine subgroups were not elevated in serum MOG Ab POS compared to serum MOG Ab NEG group. Dotted lines represent medians. The statistical analysis was performed using Kruskal Wallis test.

The CSF cytokines/chemokines which were elevated (p<0.05) in serum MOG Ab POS compared to MOG Ab NEG are presented in Fig 2 according to T and B cell subsets, and all other molecules are presented in Fig 3. Serial cytokine/chemokines were performed during relapses in one patient with MOG Ab POS demyelination that showed persistent elevation of B cell related cytokines (CXCL13, CCL19 & APRIL), IL-6 and G-CSF even during relapses (data not shown).

### Comparison of CSF cytokine/chemokine levels in MOG Ab POS group based on CSF MOG Ab positivity (Fig 4)

**Fig 4:**
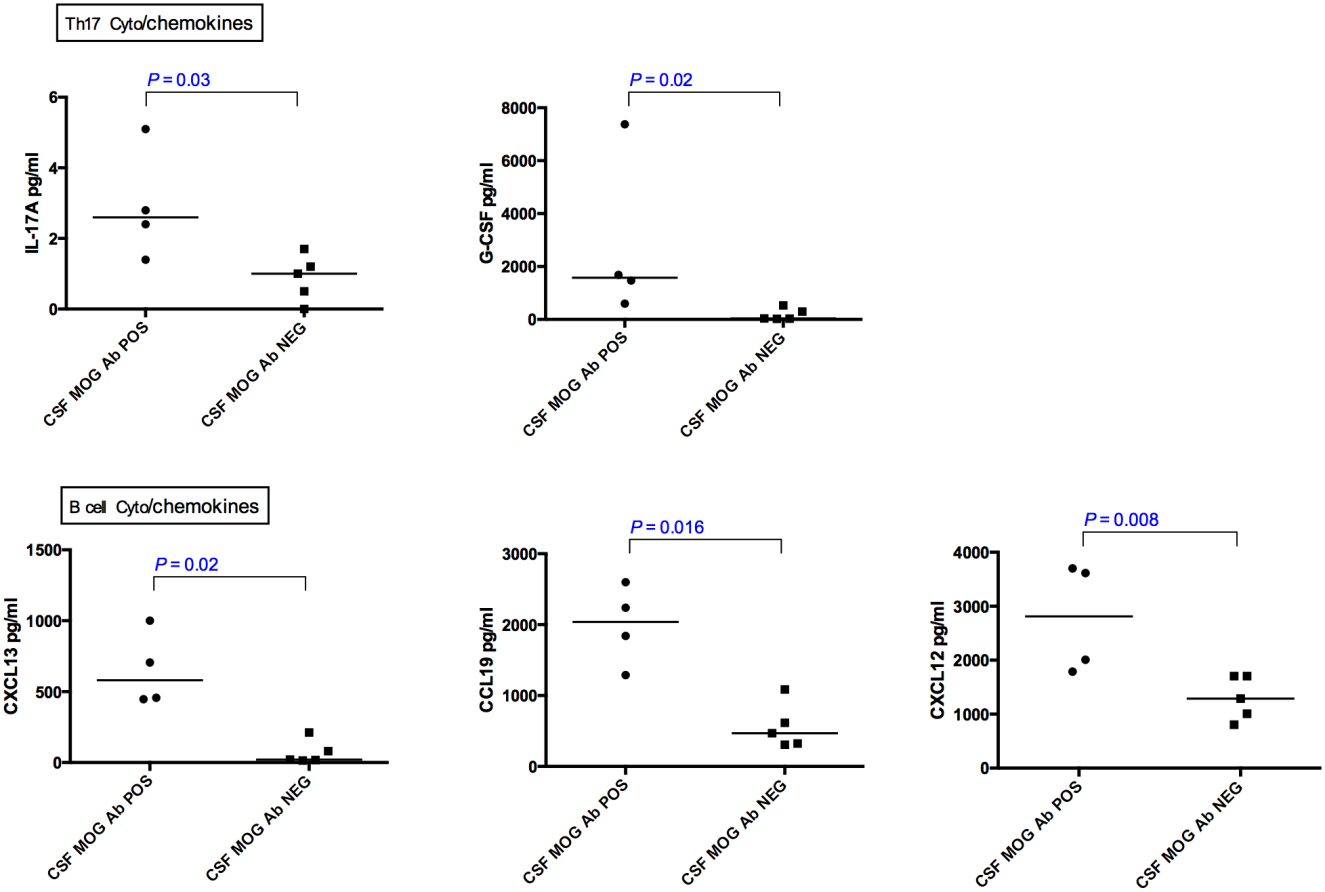
CSF cytokine/ chemokine concentrations in CSF MOG Ab POS patients and CSF MOG Ab NEG patients. B cell (CXCL13, CCL19, CXCL12,) and Th17 (G-CSF, IL-17A) cytokines/chemokines were elevated in CSF MOG Ab POS patients when compared to CSF MOG Ab NEG patients (P<0.05). Dotted lines represent medians. The statistical analysis was performed using Man Whitney’s test.

Nine of the 10 serum MOG Ab POS patients had adequate CSF available for CSF MOG antibody testing. Four of these 9 samples were positive for CSF MOG Ab, and 5 were CSF MOG Ab negative. All 34 CSF cytokines and chemokine levels were compared, of which only median concentrations of B cell (CXCL13, CCL19, CXCL12) and Th17 (G-CSF, IL-17A) cytokines were higher (p <0.05) in CSF MOG Ab POS group compared to CSF MOG Ab NEG group (Fig 4).

### Correlation between CSF cytokines/chemokines and clinical parameters in patients with serum MOG Ab POS and MOG Ab NEG

**Fig 5:**
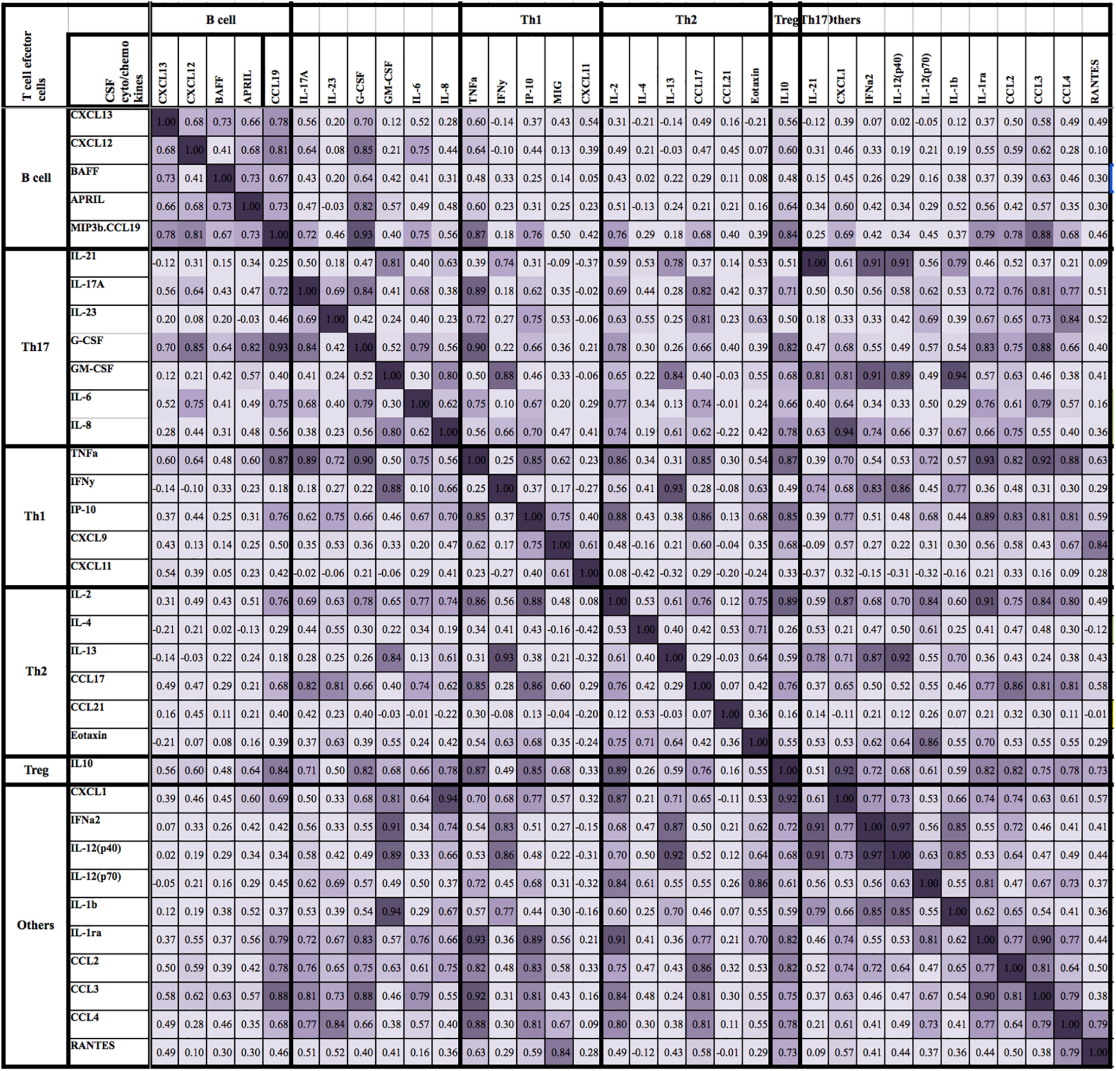
Correlation between CSF cytokine and chemokine levels in patients with MOG Ab POS demyelination. This figure shows that the majority of B cell cyto/chemokines correlate with each other and with Th17 molecules (IL-17A, IL-6 and G-CSF) and IL-10, but do not show any association with Th1 (except TNF-α) and Th2 related cytokines/chemokines. The above heat map is graded based on Spearman rank correlation* 0.0 to 0.03, negligible correlation; 0.3 to 0.5, low positive correlation; 0.50 to 0.70, moderate correlation; 0.70 to 0.90, high correlation; 0.9 to 1, very high correlation). *The negative correlations were all moderate or weaker so have been coded the same as the “negligible” correlations.

There were no correlations of cytokines/chemokines with disability outcome using modified Rankin scale at admission and follow up. Most cytokine/chemokine levels decreased with longer duration of CSF sampling after onset of neurological symptoms. The majority of B cell cyto/chemokines correlated with each other and with Th17 molecules (IL-17A, IL-6 and G-CSF) and IL-10, but did not show any relation with Th1 (except TNF-α) and Th2 related cytokines/chemokines (presented as heat map in Fig 5).

## Discussion

Despite the increasingly available literature on MOG antibodies in CNS demyelination syndromes, the pathophysiology of the inflammatory processes underlying MOG antibody associated demyelination remains to be fully elucidated. This is the first study to report elevation of CSF cytokines and chemokines in MOG Ab-associated demyelination, and therefore investigate additional immunopathological aspects other than autoantibody-associated mechanisms.

We ensured that patient groups were clinically similar including a similar proportion of ADEM, and similar timing of CSF sampling. Although there were slightly larger numbers of ADEM patients in the MOG Ab POS group, the findings were similar when only ADEM patients were compared (data not shown). As previously reported, the serum MOG Ab POS patients in our cohort were younger at presentation, had higher systemic inflammatory markers (ESR), higher CSF cell count, CSF protein, CSF IgG levels, higher lesion load on MRI and more often had relapses and minor neurological deficits at follow-up which suggests that MOG Ab associated demyelination is a more inflammatory CNS syndrome than MOG Ab negative demyelination [2–4, 12, 25].

We investigated a comprehensive array of cytokines/chemokines in our patients with seropositive and seronegative MOG Ab associated demyelination. Although Th1 and Th2 cytokines were often elevated in both MOG Ab POS and MOG Ab NEG groups compared to controls, there was no significant difference between the serum MOG Ab POS and MOG Ab NEG groups except for TNF-α. Interestingly, TNF-α has been shown to be produced by both Th1 and Th17 cells in *in vitro* studies [26].

All of the measured CSF B cell associated chemo/cytokines (CCL19, CXCL13, CXCL12, BAFF, and APRIL) were higher in the serum MOG Ab POS group compared to MOG Ab NEG group. These chemo/cytokines play an important role in recruitment, clonal selection and expansion of B cells [27–31]. In addition, CXCL13 plays a role in the formation of ectopic lymphoid tissues and facilitates migration of Th 17 cells, whereas BAFF and APRIL are important for the development and survival of B cells [32–34]. CSF CXCL13, APRIL, and BAFF levels have been shown to be elevated in autoimmune disorders, including anti-NMDA receptor encephalitis, opsoclonus myoclonus ataxia syndrome, NMO, multiple sclerosis and optic neuritis. Previous studies reported that CSF CXCL13 correlates with intrathecal immunoglobulin production and presence of CSF plasma cells or plasmablasts better than any other B cell—attracting chemokines [35–39].

Th17 related cytokines/chemokines, predominantly IL-6 and G-CSF, were significantly elevated in serum MOG Ab POS group compared to MOG Ab NEG group. Recently IL-6 was reported to correlate with MOG antibody titers in paediatric patients with monophasic acquired demyelination syndromes [40]. IL-6 activates B cells and is dysregulated in a variety of autoimmune disorders [41] including NMO [20] and ADEM [42]. In addition, IL-6 is a critical switch factor that diverts antigen activated naïve T cells towards proinflammatory Th17 cell lineage in the presence of TGF-β and IL-23, which in turn aggravates inflammation by producing additional cytokines/chemokines [26]. Granulocyte colony-stimulating factor (G-CSF) production is induced by Th17 cells, which stimulate survival, proliferation, and differentiation of neutrophils [43, 44]. Although this data supports a possible role of Th17 immunity, in view of the pleiotropic nature of IL-6 and G-CSF it is possible that these cytokines are produced by cells other than Th17 cells. The persistent elevation of the cytokines/chemokines CXCL13, G-CSF, IL-6, APRIL, CCL19 during relapses in one patient with relapsing MOG Ab POS demyelination raises the possible utility of these markers in monitoring disease. The elevation of CSF IL-10 and IL-1ra in serum MOG Ab POS group compared to MOG Ab NEG group may suggest an *in vivo* regulatory response to counteract proinflammatory cytokines [45].

Even though there is emerging evidence that MOG antibody associated demyelination is an antibody-mediated disease, there is increasing evidence that active cooperation between Th17 and B cells against MOG antigen is necessary for immunopathogenicity, as shown in EAE mouse model studies [26, 46–48]. The correlation between different B cell cyto/chemokines and with Th17 molecules (IL-17A, IL-6 and G-CSF) and IL-10 observed in our study suggests possible interactions *in vivo* (Fig 5). Therefore it is likely that multiple inflammatory cell types play an important pathogenic role in MOG antibody associated demyelination, and our study has suggested that MOG associated demyelination is immunopathologically complex. The predominant elevation of CSF B cell and some Th17 and neutrophil related cytokines/chemokines in serum and CSF MOG Ab positive demyelination is similar to what has been described in NMO [18, 20, 49, 50]. To date there are no cytokine/chemokine studies in adults with MOG Ab POS demyelination syndromes. In view of different clinical associations, it is possible that adult patients with MOG Ab may have a different cytokine profile to children.

Even though earlier studies showed no differences between outcomes between MOG Ab POS and NEG cases, we are increasingly learning about MOG Ab POS cases that have relapses and long-term neurological sequelae [5, 7, 9, 10]. In our series MOG Ab POS cases had a higher lesion load on MRI, and more often had relapses and deficits on follow up. Overall, our findings have important clinical implications in order to optimize patients’ treatment. For example, IL-6 may have some roles in the pathogenesis of MOG Ab demyelination and the anti-IL-6 pathway could be a promising target for new pharmacological treatments of steroid resistant MOG Ab associated demyelination, as has recently been shown in NMO [51]. Due to the predominant humoral pathogenic mechanisms, it seems rationale to treat MOG antibody associated demyelination with immune suppression including steroids, plasma exchange, or a B-cell-directed therapy, similar to NMO [52, 53]. However it is possible that different patients may have different dominant immunopathogenic mechanisms leading to variability in therapeutic response.

The limitations of our study are the fact our MOG Ab POS and NEG groups were not identical in clinical and demographic parameters, despite our efforts to balance our cohorts according to demyelination phenotype. The sample size was small in each group and the current study was confined to the paediatric group only. Our control group aimed to generate normative data by using non-inflammatory neurological controls and future studies should compare demyelination syndromes with infectious and autoimmune encephalitis. The storage of samples for prolonged periods can result in theoretical degradation of cytokines/chemokines, although we did not clearly observe that (data not shown). And finally, we could not perform comparisons between serum and CSF samples to determine whether the cytokine/chemokines are predominantly produced intrathecally or peripherally, due to the lack of available stored frozen serum samples.

In summary, our study points to a role of predominant humoral immunity and possible role of Th17 cells and neutrophils in MOG antibody associated demyelination, which strengthens the hypothesis that MOG antibody defines a separate immunopathological syndrome with dominant humoral autoimmunity.

## Supporting Information

S1 File Table A. Details of clinical syndrome, treatment and outcome of MOG Ab POS and NEG patients with acute demyelination episode. Table B. Comparison of radiological abnormalities between MOG Ab POS and MOG Ab NEG demyelination groups.
(DOCX)

## Abbreviations

MOG: Myelin oligodendrocyte glycoprotein antibody
ADEM: Acute Disseminated Encephalomyelitis
TM: Transverse Myelitis
NMO: Neuromyelitis Optica
NMOSD: Neuromyelitis Optica Spectrum Disorders
MRI: Magnetic Resonance Imaging
CSF: Cerebrospinal Fluid
Ab: Antibody
POS: Positive
NEG: Negative
EAE: Experimental Allergic Encephalomyelitis
