# Supplementary material for: B Cell, Th17, and Neutrophil Related Cerebrospinal Fluid Cytokine/Chemokines Are Elevated in MOG Antibody Associated Demyelination
Source: PLoS One. 2016 Feb 26;11(2):e0149411. doi: 10.1371/journal.pone.0149411 (PMC4769285; doi:10.1371/journal.pone.0149411)
Supplement: S1 File — Table A. Details of clinical syndrome, treatment and outcome of MOG Ab POS and NEG patients with acute demyelination episode. Table B. Comparison of radiological abnormalities between MOG Ab POS and MOG Ab NEG demyelination groups. (DOCX) [file pone.0149411.s001.docx]

**Table A: Details of clinical syndrome, treatment and outcome of MOG Ab POS and NEG patients with acute demyelination episode**

| Case no | Initial diagnosis | Age at first episode/ sex | Treatment: acute phase | Progress MRI (FU)(mo) | Outcome (MRS) (FU) (mo) | Relapse | Neurological deficits | Treatment in FUP |
| --- | --- | --- | --- | --- | --- | --- | --- | --- |
| MOG Ab POS | | | | | | | | |
| 1 | ADEM | 7.8 /F | IVMP and steroid wean | Moderate (1.5) | 0 (1) |  | No deficits (too early) | NA |
| 2 | ADEM | 5.1/M | IVMP and steroid wean | Minor (12) | 2 (24) | Relapses (ADEM, 2 E) | Fatigue, concentration and memory diff | MMF + alternate day P |
| 3 | ADEM, Symp s/o SC | 2.7/M | IVMP and steroid wean | Exvacuo dilatation, optic atrophy (52) | 2 (52) |  | Fine motor incoordination | NA |
| 4 | ADEM | 2/M | IVMP and steroid wean | ND | 2 (39) |  | Worsening of pre existing learning diff | NA |
| 5 | ADEM | 3.1/M | IVMP and steroid wean | Compl resol (2.5) | 1 (42) |  | Slightly increased tone | NA |
| 6 | TM+ Asympt brain lesions | 11.2/M | IVMP and steroid wean | Minor (Br), Compl resol (Sp) (7) | 2 (2) |  | Fatigue, decreased vibration in toes | NA |
| 7 | TM+ ON + Asympt brain lesions | 14/F | IVMP, PE, and steroid wean | Minor (Br), Compl resol (Sp) (11) | 2 (8) |  | Residual chronic pain in limbs | Monthly IVIG*3, alternate day P |
| 8 | ADEM | 4.5/F | IVMP and steroid wean | Moderate (9) | 0 (6) | Relapses (ADEM, 3E) | No deficits | MMF +alternate day P |
| 9 | ADEM | 4.9/M | IVMP and steroid wean | Compl resol (11) | 3 (14) |  | Learning, behavioural, attention diff | NA |
| 10 | ADEM, Symp s/o SC | 3/F | IVMP and steroid wean | Compl resol (Br) and (Sp) (2) | 0 (18) |  | No deficits | NA |
| MOG Ab NEG | | | | | | | | |
| 11 | ADEM | 9.3/M | IVMP and steroid wean | Compl resol (3) | 0 (5) |  | No deficits | NA |
| 12 | ADEM | 10.6/F | IVMP and steroid wean | Minor (48) | 0 (19) | Relapse (ADEM, 1E) | No deficits | NA |
| 13 | ADEM | 7/M | None | Compl resol (3) | 0 (12) |  | No deficits | NA |
| 14 | ADEM | 5.5/M | IVMP, IVIG and steroid wean | ND | 2 (24) |  | Left sided weakness | NA |
| 15 | ADEM | 7/M | IVMP and steroid wean | Compl resol (24) | 3 (54) |  | Learning, behavioural, attention diff | NA |
| 16 | TM | 7/F | IVMP, IVIG and steroid wean | ND | 2 (64) |  | Significant left arm weakness & subluxation of left shoulder | NA |
| 17 | TM | 10/F | IVMP, IVIG and steroid wean | ND | 0 (8) |  | No deficits | NA |
| 18 | TM | 13/F | IVMP, IVIG and steroid wean | Compl resol (Sp) (6) | 1 (36) |  | Mild sensory abnormalities | NA |
| 19 | TM | 12/M | IVMP and steroid wean | Compl resol (Sp) (1.5) | 0 (14) |  | No deficits | NA |

**Abbreviations:** Sympt, Clinical symptoms; Asympt, Asymptomatic; IVMP, Intravenous Methyl Prednisolone; PE, Plasma exchange; IVIG, Intravenous Immunoglobulins, MMF, Mycophenolate; Prednisolone, P; Compl resol; Complete resolution; Brain, Br; Sp, Spine; ND, Not Done; MRS, Modified Rankin scale; E, Episode; difficulties, diff; FU, Follow Up; mo, months: NA, Not Applicable

**Table B: Comparison of radiological abnormalities between MOG Ab POS and MOG Ab NEG Acute Disseminated Encephalomyelitis*.**

|  | **MOG Ab POS (n=8)** | **MOG Ab NEG (n=5)** |
| --- | --- | --- |
| **Supratentorial white matter** |  |  |
| Juxtacortical | 4/8 | 1/5 |
| Non-juxtacortical | 8/8 | 4/5 |
| Non-periventricular white matter | 8/8 | 4/5 |
| Corpus callosum | 4/8 | 0/5 |
| Periventricular white matter | 2/8 | 0/5 |
| ***Score (1-5)*** | 3.1 | 1.8 |
| Thalamus | 4/8 | 1/5 |
| Basal ganglia | 6/8 | 2/5 |
| Brainstem | 4/8 | 4/5 |
| Cerebellum | 3/8 | 1/5 |
| LETM (longitudinal extensive transverse myelitis >3) | 4/7 | 4/4 |
| ***Optic nerve*** | 2/8 | 0/5 |
| Orbital View (Yes/No) | 0/8 | 0/5 |
| **Configuration of white matter lesions** |  |  |
| Blurred margins/Hazy (>80%) | 7/8 | 5/5 |
| ***Large (>2 cm on the axial sequence)*** | 3/8 | 1/5 |
| Diffusion restriction | 0/8 | 0/5 |
| ***Contrast-enhancing*** | 6/8 | 0/5 |
| Bilateral lesions | 8/8 | 4/5 |
| **Atypical MRI features** |  |  |
| Only one anatomical MRI area affected (Location) | 0/8 | 2/5 |
| Diffuse white matter involvement without clear lesional character | 1/8 | 1/5 |
| Small white matter lesions (<2 cm) | 5/8 | 3/5 |
| ***T1-hypointense lesions*** | 5/8 | 0/5 |
| Well defined borders | 2/8 | 1/5 |
| Lesions perpendicular to the corpus callosum | 0/8 | 0/5 |
| Periventricular | 2/8 | 0/5 |
| Cortical lesions | 1/8 | 2/5 |
| MS like features (well-defined lesions together with a periventricular or perpendicular location to the long axis of the corpus callosum) | 0/8 | 0/5 |
| Other Regions | 2/8 | 0/5 |
| Number of atypical features (Mean) | 1.8 | 1.8 |
| **MRI outcome (Brain)** |  |  |
| Complete resolution | 4/7 | 3/4 |
| Minor residuals (few remaining T2 signal changes, but much improved) | 1/7 | 1/4 |
| Moderate residuals (only minor improvement of T2 signal changes) | 2/7 | 0 |
| Marked residuals (atrophy) | 0 | 0 |
| **MRI outcome (Spine)** |  |  |
| Complete resolution | 2/2 | 2/3 |
| Minor residuals (few remaining T2 signal changes, but much improved) | 0 | 1/3 |

***The radiological differences are underlined**
